# Rapidly Progressive Apical Hypertrophic Cardiomyopathy: Not Everything is What It Seems

**DOI:** 10.14797/mdcvj.1386

**Published:** 2024-06-21

**Authors:** Ahmed K. Mahmoud, Juan M. Farina, Kamal Awad, Milagros Pereyra, Isabel G. Scalia, Mohammed Tiseer Abbas, Timothy Barry, Said Alsidawi, Chadi Ayoub, Reza Arsanjani

**Affiliations:** 1 Mayo Clinic, Phoenix, Arizona, US

**Keywords:** cardiomyopathy, physical exertion, electrocardiography, syncope, dyspnea

## Abstract

Apical hypertrophic cardiomyopathy (HCM) is a rare variant of HCM. A 43-year-old female with a past medical history significant for hypertension and kidney transplantation presented with recurrent syncopal episodes and dyspnea on exertion. Electrocardiogram showed characteristic diffuse giant T-waves inversion, and cardiac magnetic resonance showed HCM with circumferential apical thickening. This case highlights the rapid development of apical HCM and its challenging diagnostic characteristics.

## Introduction

Apical hypertrophic cardiomyopathy (HCM) is a morphological subtype of HCM that is characterized by increased apical wall thickness and precordial deep symmetrical T-wave inversion on electrocardiogram (ECG).^1^ Notably, it has been proposed to be a disease with early clinical manifestations consistent with the age-related penetrance of traditional HCM and has a predilection for middle-aged men.^1,2^ Accurate diagnosis of apical HCM can be challenging due to technical difficulties visualizing the apical endocardial borders by conventional transthoracic echocardiography (TTE), heterogeneous clinical manifestations, and lack of established diagnostic criteria.^2^ Compared to HCM with septal morphologic subtypes, apical HCM is more sporadic, has a later onset, has less-frequently detected genetic variants, and may be associated with apical aneurysm or pouch, an adverse prognostic feature. Additionally, there is less robust evidence for prognosis and mortality risk in this population.^3^ We are reporting this current case to highlight the challenging diagnostic characteristics of this disease and its potential for rapid development.

## Case Report

**Figure 1:**
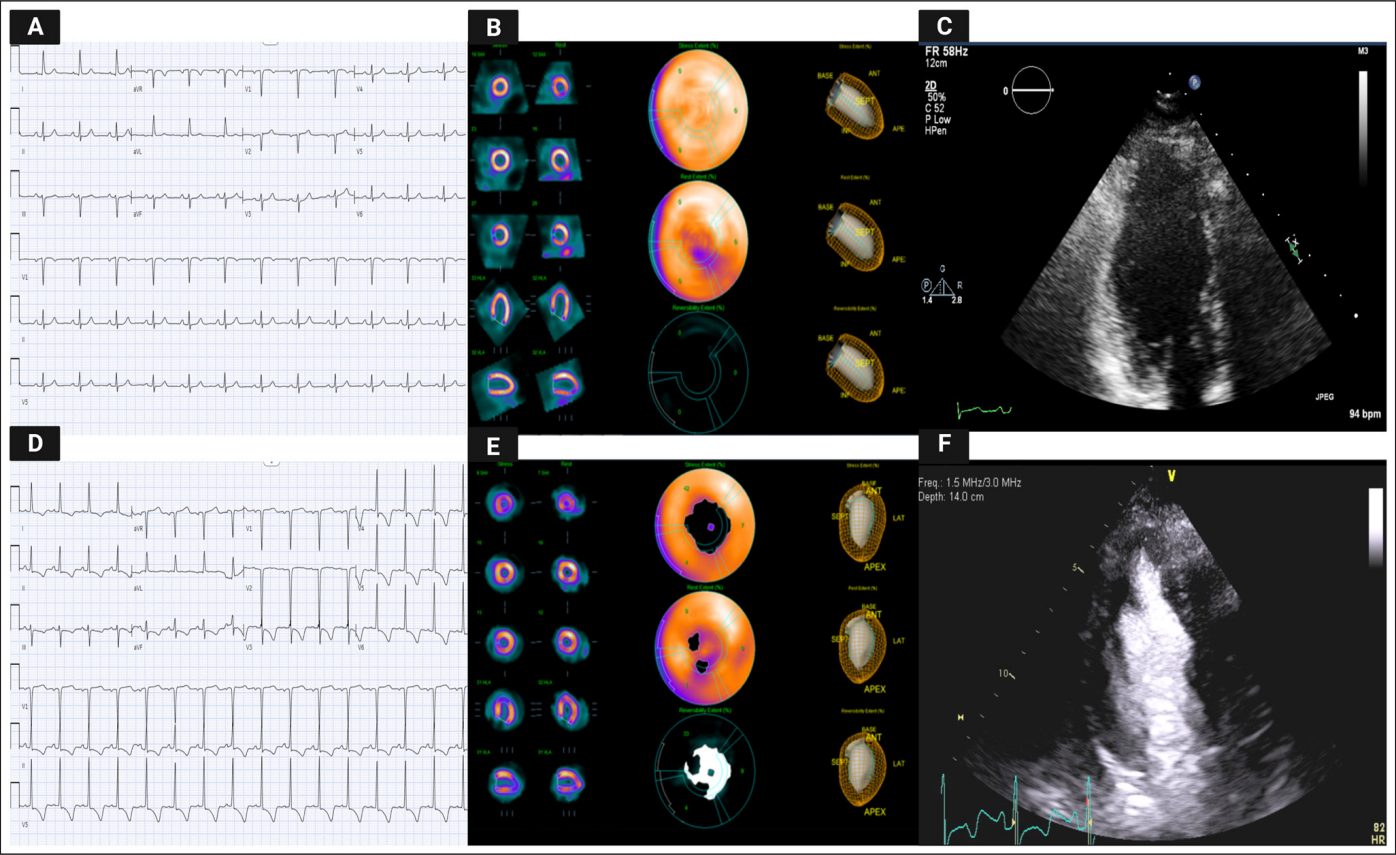
Illustration of the comparison between multi-imaging modalities in a 1-year follow-up period. **(A)** Previous electrocardiogram (ECG) showed no abnormalities compared to the ECG at current presentation **(D)**, which showed diffuse T wave inversion. **(B, E)** Myocardial perfusion studies demonstrate side-by-side comparison of new ischemia involving the distal inferior and apical segments. **(F)** Transthoracic echocardiography show new apical hypertrophy in parasternal short axis with enhancing agent compared to the previous study **(C)**.

A 43-year-old female with a past medical history significant for hypertension and kidney transplantation presented with recurrent syncopal episodes and dyspnea on exertion. Notably, 1 year prior to the current presentation, the patient had a normal 12-lead ECG (Figure 1A), a negative nuclear stress test (Figure 1B), and a TTE showing basal septal hypertrophy (Figure 1C; Video 1). She had no known family history of HCM, and genetic testing did not identify any known HCM gene.

**Video 1:** Apical four-chamber echocardiography view before diagnosis showing no significant abnormalities; see also at https://youtu.be/NAWtaBxa1lw.

**Figure 2:**
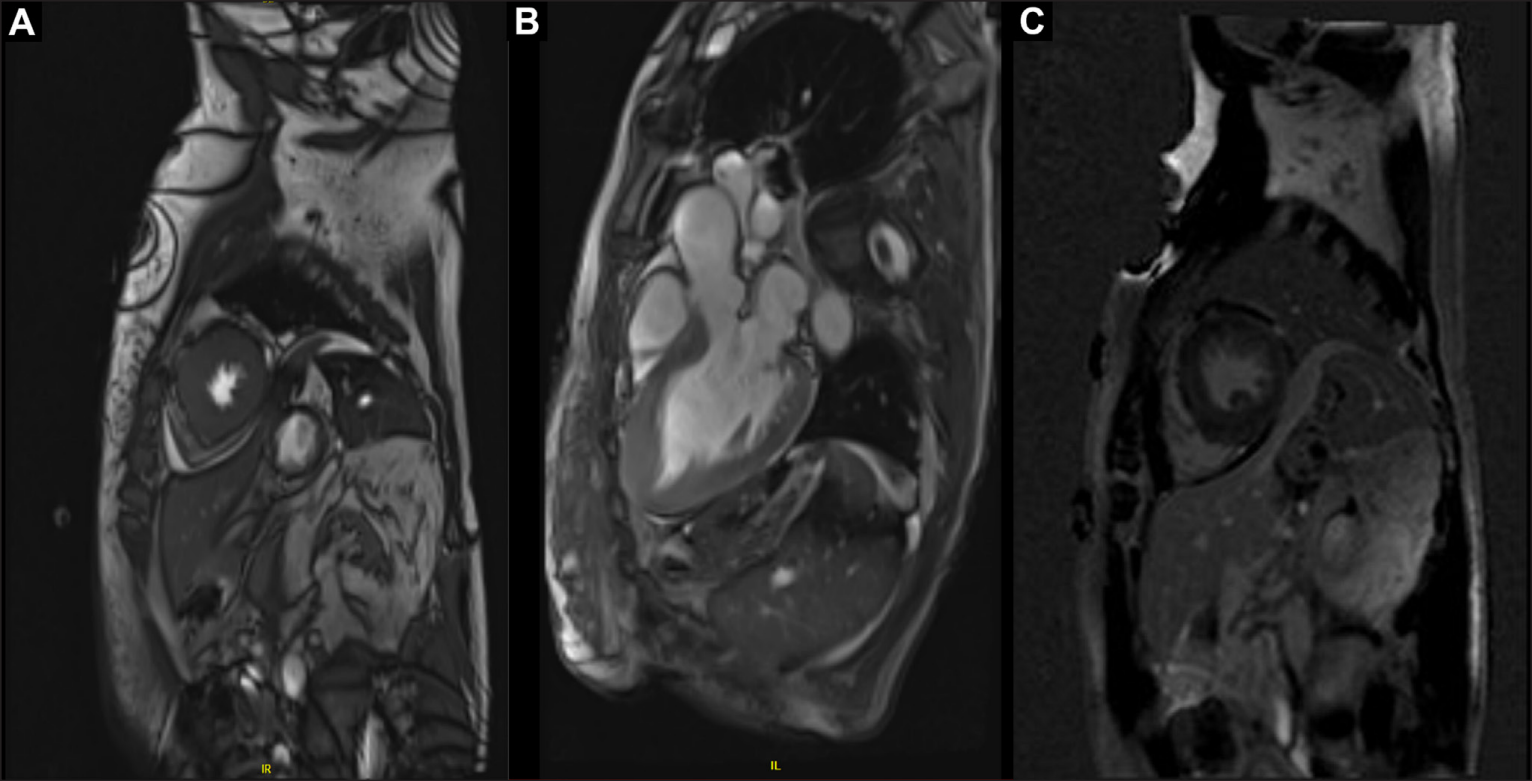
Cardiac magnetic resonance study demonstrates apical hypertrophic cardiomyopathy with the presence of fibrosis in the apical region. **(A)** Short axis image 3-chamber view at the left ventricular apical level **(B)** and within the apical interventricular septum and inferior wall. **(C)** Roughly 5% patchy mid-myocardial areas of minimal late gadolinium enhancement can be identified.

With her current presentation, ECG showed new diffuse giant T-waves inversion, which raised suspicion for cardiac ischemia (Figure 1D). Therefore, a nuclear stress test was repeated and demonstrated reversible perfusion defects in the left ventricular apical and periapical areas (Figure 1E). She subsequently underwent coronary angiography that showed nonobstructive coronary lesions. Repeat TTE demonstrated apical hypertrophy as well as prominent papillary muscle that resulted in mid-ventricle crowding (Figure 1F; Video 2, 3); however, no regional wall motion abnormalities were seen. Cardiac magnetic resonance (CMR) was performed and showed apical HCM with circumferential apical thickening measuring 17 mm (Figure 2A, B; Video 4). Within the apical septum, minimal fibrosis (qualitatively 5% total left ventricular myocardial volume) was observed(Figure 2C). No associated apical aneurysm or pouch was seen. Accordingly, the diagnosis was revised to apical HCM variant.

**Video 2:** Apical four-chamber echocardiography view after diagnosis showing the apical hypertrophy; see also at https://youtu.be/nC0sTpOcq9c.

**Video 3:** Apical two-chamber echocardiography view with contrast after diagnosis showing the apical hypertrophy; see also at https://youtu.be/x6nKidbm-us.

**Video 4:** Cardiac magnetic resonance study demonstrates apical hypertrophic cardiomyopathy; see also at https://youtube.com/shorts/nSbHeF8F2Wg.

## Discussion

This case highlights very rapid development of ECG imaging and clinical manifestations of apical HCM in a female previously thought to have septal hypertrophy secondary to hypertension with renal disease. Although previously asymptomatic, she presented with recurrent syncope and new ECG changes that led to initial evaluation to rule out coronary artery disease. After excluding significant coronary stenosis, a diagnosis of apical HCM was made with the aid of multimodality imaging techniques. Remarkably, several relatively recent ECG and TTEs had been performed but did not show an apical HCM pattern.

The late onset and insidious nature of apical HCM compared to other HCM variants has been previously described, with ECG changes preceding imaging and clinical manifestations of the disease by many years. However, this case demonstrates that the progression of symptoms as well as morphological and imaging manifestations can occur rapidly, thus highlighting that HCM can have variable penetrance and expression, and hypertrophy in some may develop later in life. The use of genetic testing in this condition should be interpreted with caution since these tests have been reported to be negative in approximately 75% to 90% of apical HCM cases.^3,4^ Diagnosis can also be challenging with potential differential diagnoses that may explain wall thickening, such as the presence of hypertension and advanced renal failure. Use of ultrasound-enhancing agents may enhance endocardial definition and help better define wall thickness (Video 3). CMR is invaluable in better defining morphology and fibrosis pattern.^5,6^

This case illustrates how a new diagnosis of apical HCM was confirmed after new onset of symptoms. The rapid changes in imaging studies over a short period of time emphasize the importance of conscientious clinical suspicion and close follow-up.

## Conclusions

Apical HCM is an infrequent disease with symptoms and ECG abnormalities that can mimic coronary ischemia.^7^ It can present both with gradual or rapid symptoms and morphological progression in middle-aged patients with no clear history of cardiovascular symptoms and no obvious prior suspicion of HCM. Clinical judgement, close follow-up, and multimodality imaging including CMR and ultrasound-enhancing agents are critical in establishing the diagnosis.
